# Whole-genome sequencing of *Mesorhizobium huakuii* 7653R provides molecular insights into host specificity and symbiosis island dynamics

**DOI:** 10.1186/1471-2164-15-440

**Published:** 2014-06-06

**Authors:** Shanming Wang, Baohai Hao, Jiarui Li, Huilin Gu, Jieli Peng, Fuli Xie, Xinyin Zhao, Christian Frech, Nansheng Chen, Binguang Ma, Youguo Li

**Affiliations:** State Key Laboratory of Agricultural Microbiology, Huazhong Agricultural University, Wuhan, Hubei P. R. China; Center for Bioinformatics, School of Life Science and Technology, Huazhong Agricultural University, Wuhan, Hubei P. R. China; Department of Molecular Biology and Biochemistry, Simon Fraser University, Burnaby, British Columbia Canada

**Keywords:** *Mesorhizobium huakuii* 7653R, Genome sequencing, Comparative analysis, Host specificity, Symbiosis island

## Abstract

**Background:**

Evidence based on genomic sequences is urgently needed to confirm the phylogenetic relationship between *Mesorhizobium* strain MAFF303099 and *M. huakuii*. To define underlying causes for the rather striking difference in host specificity between *M. huakuii* strain 7653R and MAFF303099, several probable determinants also require comparison at the genomic level. An improved understanding of mobile genetic elements that can be integrated into the main chromosomes of *Mesorhizobium* to form genomic islands would enrich our knowledge of how genome dynamics may contribute to *Mesorhizobium* evolution in general.

**Results:**

In this study, we sequenced the complete genome of 7653R and compared it with five other *Mesorhizobium* genomes. Genomes of 7653R and MAFF303099 were found to share a large set of orthologs and, most importantly, a conserved chromosomal backbone and even larger perfectly conserved synteny blocks. We also identified candidate molecular differences responsible for the different host specificities of these two strains. Finally, we reconstructed an ancestral *Mesorhizobium* genomic island that has evolved into diverse forms in different *Mesorhizobium* species.

**Conclusions:**

Our ortholog and synteny analyses firmly establish MAFF303099 as a strain of *M. huakuii*. Differences in nodulation factors and secretion systems T3SS, T4SS, and T6SS may be responsible for the unique host specificities of 7653R and MAFF303099 strains. The plasmids of 7653R may have arisen by excision of the original genomic island from the 7653R chromosome.

**Electronic supplementary material:**

The online version of this article (doi: 10.1186/1471-2164-15-440) contains supplementary material, which is available to authorized users.

## Background

Rhizobia are nitrogen-fixing soil bacteria constituting around 100 known species classified into 13 genera [1, 2]. *Mesorhizobium*, whose growth rate is intermediate between that of genera *Rhizobium* and *Bradyrhizobium*, is one of the largest genera; it presently comprises 24 species found primarily in Asia, Europe, the Mediterranean region, and Africa [2, 3]. *Mesorhizobium huakuii* and *M. loti* were two of the first species identified in the genus. The first known strain of *M. huakuii* was isolated from a winter-growing green manure crop, *Astragalus sinicus,* in Hubei, China in the 1940s by Huakui Chen [4], and was initially named *Rhizobium huakuii* by Wenxin Chen [5]. *Rhizobium huakuii* was later classified into *Mesorhizobium* gen. nov. and consequently renamed *M. huakuii*[6]. *M. huakuii* is a narrow-host-range rhizobium: it only induces indeterminate-type nitrogen-fixing nodules on the roots of *A. sinicus*, an economically important forage and green manure crop grown throughout eastern Asia in winter. The *M. huakuii* strain 7653R has been studied extensively and has been applied in sustainable agriculture for many years [7–9]. To facilitate comparative genomic investigation of the mechanism underlying this strain’s symbiosis and its host-plant molecular interactions, the first specific aim of our research was to sequence, assemble, and annotate the entire genome of 7653R.

The first completely sequenced *Mesorhizobium* strain was *M. huakuii* bv. *loti* MAFF303099, initially considered a strain of *M. loti*[10]. Comparative sequence analysis of additional conserved genes (including 16S rRNA, *glnA*, *glnII*, and *recA*) have suggested instead a closer phylogenetic relationship with strains of a different species, *M. huakuii*, prompting the hypothesis that MAFF303099 is a strain of *M. huakuii*[11]. Whole-genome sequencing of native *M. loti* strain R7A by the JGI GEBA project and various research findings related to R7A, such as genomic island mobility [12], the NifA-RpoN regulon and its symbiotic activation [13], and the role of the type-IV secretion system in genomic islands [14, 15], have provided a suitable reference strain and basis for the genomic comparison in this study. Consequently, our second goal was to determine whether genome-wide evidence supports the hypothesized assignment of MAFF303099 to *M. huakuii*.

Although MAFF303099 and 7653R may both be strains of the same species—*M. huakuii*, they display drastically different host preferences. Strain 7653R forms specific symbiosis with *A. sinicus*, whereas MAFF303099 forms determinant-type globular nodules and performs nitrogen fixing on several host plants of *Lotus*, including *L. japonicus* and *L. corniculatus*[16]. Our third aim was thus to identify genomic signatures possibly accounting for these differential host preferences.

Nodulation and nitrogen-fixation genes show remarkably different genomic locations in different genomes. While MAFF303099 and *M. loti* R7A have their nodulation and nitrogen-fixation genes concentrated in a long DNA region called a symbiosis island on their main chromosomes [12], the corresponding genes in 7653R are located primarily on plasmids [17]. Interestingly, nodulation and nitrogen-fixation gene locations of *M. ciceri* bv. *biserrulae* WSM1271 [18], *M. australicum* WSM2073 [19, 20] and *M. opportunistum* WSM2075 [19, 21], show patterns similar to those found in MAFF303099. These similarities suggest that genome recombination events and horizontal gene transfer are frequent in rhizobia. Our final objective was to define these genomic differences with the aim of elucidating their origin.

## Results and discussion

### Complete sequencing of the *M. huakuii* 7653R genome

**Figure 1:**
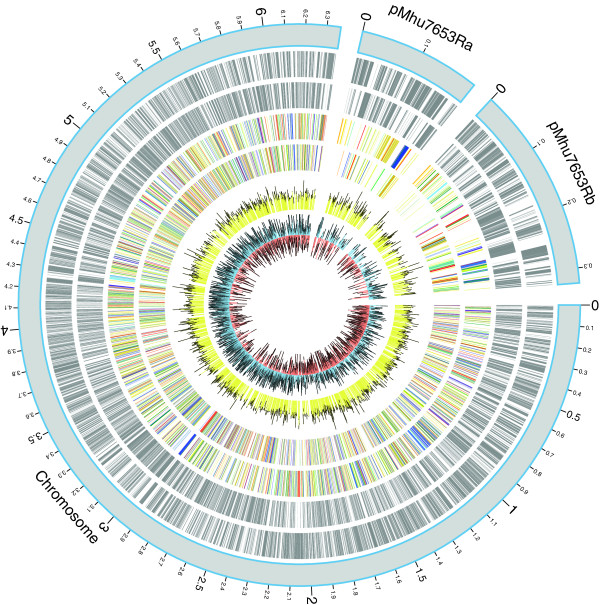
**Physical maps of the complete** ***Mesorhizobium huakuii*** **7653R genome.** Physical maps of three replicons were drawn using Circos [22]. Displayed circles from the inside outwards represent: G-C skew using a 1-kb window (ring 1), Codon Adaptation Index (ring 2), Clusters of Orthologous Groups (COGs) of proteins in a counterclockwise/clockwise direction (rings 3 and 4), predicted coding sequences transcribed in both directions (rings 5 and 6), and scale in Mb (ring 7). The position 0 represents the origin of replication in each replicon.

Our 6,881,675-bp assembly of the *M. huakuii* 7653R genome consisted of three circular replicons of 6,364,365 bp (chromosome), 193,835 bp (plasmid pMhu7653Ra), and 323,475 bp (plasmid pMhu7653Rb) (Figure 1). The average GC content of the whole genome was calculated to be 62.86%, while plasmids pMhu7653Ra and pMhu7653Rb showed slightly lower GC levels (58.0%). An overview of the GC content of the three replicons is shown on the 7653R genome physical maps (Figure 1). The main genome characteristics of 7653R and four other *Mesorhizobium* genomes (MAFF303099, WSM1271, WSM2075, and WSM2073) are summarized in Table 1. Although the five strains all belong to the same genus, they possess different numbers of plasmids: 7653R and MAFF303099 have two plasmids each, WSM1271 has only one, and WSM2073 and WSM2075 have none.

**Table 1:** **General genomic features of** ***Mesorhizobium huakuii*** **7653Rand four other mesorhizobial genomes**

	7653R	MAFF303099	WSM1271	WSM2705	WSM2073
**Size (bp)**	6,881,675	7,596,297	6,690,028	6,884,444	6,200,534
**G+C content (%)**	62.86	62.51	62.56	62.87	62.84
**Total no. of CDS**	7,205	7,281	6,264	6,508	5,792
**CDS coverage (%)**	87.9	86.0	86.2	86.0	86.2
**tRNA genes**	51	50	53	53	53
**rRNA operons**	5	6	6	6	6
**Nb (%) CDS with assigned functions**	5,459 (75.7)	5,431 (74.6)	4,573 (73.0)	4,778 (73.4)	4,466 (77.1)
**Average length of genes (bp)**	832	897	921	910	923
**Putative transposases**	76	123	12	28	9
**Tandem repeat sequences**	419	404	281	451	290
**Transcriptional regulators**	285	330	407	474	376
**Putative ABC transporters-related proteins**	675	568	561	565	585
**Putative two component systems-related proteins**	73	70	59	49	48
**Putative sigma factor-related proteins**	29	24	30	32	28

CDS: coding sequences.

Statistics for MAFF303099, WSM1271, WSM2075, and WSM2073 genomic features are based on NCBI annotations.

We predicted 7,205 protein-coding genes in the 7653R genome, a number essentially identical despite the different genome sizes to the number predicted in MAFF303099 (7,281 genes) [10]. 7653R was found to have the highest gene density among the five genomes, but have a lower ratio of genes with annotated functions, suggesting that it contains a higher ratio of genes with undefined functions. We examined the numbers and types of rRNAs and tRNAs of all five genomes predicted using the same strategy. We found that these five genomes had essentially identical numbers of rRNAs and tRNAs (Additional file 1: Table S1). However, the numbers of putative transposase genes predicted in these genomes were dramatically different (Table 1). As discussed later, this variation may have a profound differential impact on genome stability and horizontal gene transfer (HGT) events.

### Genomic evidence supporting MAF303099 as a strain of M. huakuii

MAFF303099 has been hypothesized to be a strain of *M. huakuii* on the basis of comparative analysis of a few conserved genes in MAFF303099 and *M. huakuii* strains [11]. The availability of genome sequences of both strains has enabled us to re-examine their phylogenetic relationship.

#### Genome-wide orthologs

**Figure 2:**
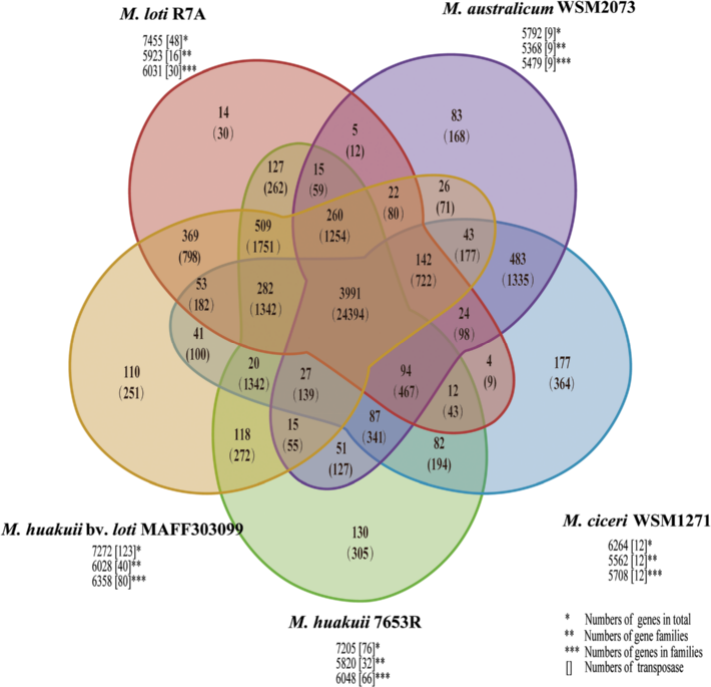
**Core and accessory genome analyses of** ***Mesorhizobium*** **strains.** The numbers of orthologous groups and related genes found in each intersection are shown. The numbers of genes found in related strains for each intersection are shown in parentheses. The numbers of transposase genes are shown in square brackets. Areas are not in scale.

**Figure 3:**
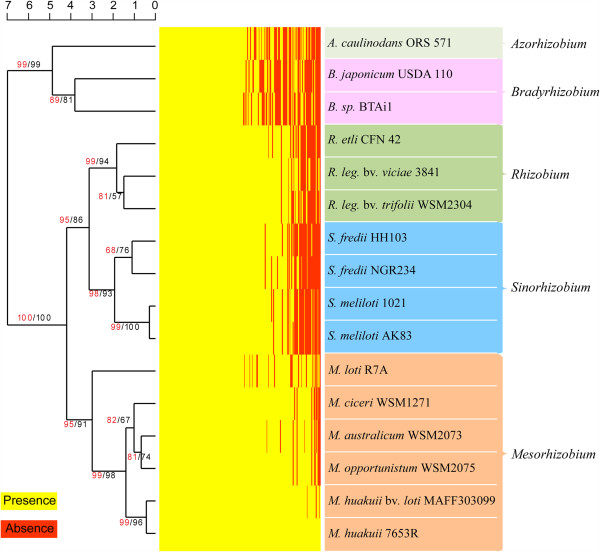
**Hierarchical clustering analysis of rhizobia based on a heat map of 210 genes chosen from** ***Mesorhizobium*** **core genes.** Gene homologs were chosen on the basis of BLASTP results (*E*-value ≤1 ×10^−5^; identity ≤ 35%). Homolog presence and absence are indicated by yellow and red, respectively. The five genera for which multiple genomes were available are indicated in different colors. The numbers on the dendrogram represent bootstrap values.

We identified a set of 7,414 orthologous groups among five *Mesorhizobium* genomes (7653R, MAFF303099, R7A, WSM1271, and WSM2073). Of these groups, 3,991 (54%) were found to be conserved across all five genomes, with each group represented by at least one gene in each of the five genomes. We termed this subset of orthologous groups the core genome of *Mesorhizobium*. An additional 805 (11% of orthologous groups) were observed to be present in four of the five genomes (Figure 2). The remaining orthologous groups (28% or 2,104) occurred in two or three genomes. Similar numbers and proportions of proteins predicted in 7653R (4,073; 57.5%) and MAFF303099 (4,064; 57.1%) were present in the core genome, whereas 54.5% (4,064) of predicted proteins in R7A were present in the core genome. Among all pair-wise comparisons, the 7653R-MAFF303099 pair was found to share the most abundant orthologous proteins, followed by the R7A-MAFF303099 and WSM1271-WSM2073 pairs; this ordering suggests that MAFF303099 has a closer phylogenetic relationship to 7653R than to R7A, WSM1271, and WSM2073. From the 4,073 7653R core genes, we randomly chose 210 single-copy genes (Additional file 2: Table S2) and performed hierarchical clustering analysis [23] based on their presence or absence in 16 representative rhizobial species. The clustering results also revealed a closer phylogenetic relationship between 7653R and MAFF303099 (Figure 3), further supporting MAFF303099 as a strain of *M. huakuii*.

#### Synteny analysis

**Figure 4:**
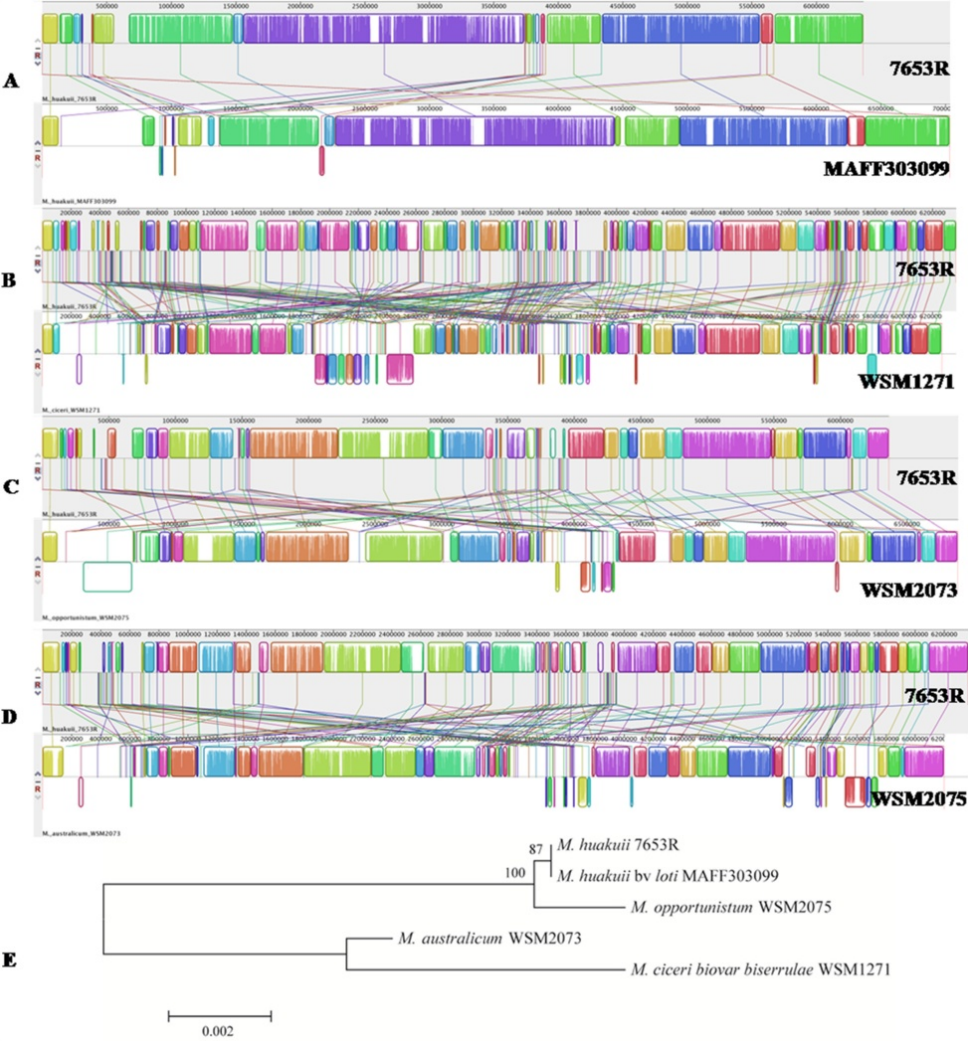
**Chromosomal alignment of five mesorhizobial species (A–D) visualized using Mauve and a phylogenetic tree (E).** Chromosomal alignments for 7653R and MAFF303099 **(A)**, 7653R and WSM1271 **(B)**, 7653R and WSM2073 **(C)**, and 7653R and WSM2075 **(D)** were created using Mauve software. Each colored block represents a synteny block and is internally independent from genomic rearrangement. White regions correspond to unaligned sequences that likely contain sequence elements specific to a particular genome. Blocks below the center line indicate regions that are aligned in the reverse complement (inverse) orientation. Phylogenetic tree **(E)** created by Mauve based on DNA sequence consistency.

The above analysis of orthologs suggested that MAFF303099 is phylogenetically more closely related to *M. huakuii* strain 7653R than to strains of other species. We further hypothesized that *M. huakuii* strain 7653R and MAFF303099 share larger synteny blocksbetween them than with the other three strains. To test this hypothesis, we carried out a synteny analysis of five strains (7653R, MAFF303099, WSM1271, WSM2073, and WSM2075) using a few complementary approaches. For convenience of comparison, we considered *dnaA* to be the start position of the main chromosome and *repABC* to be the start position of plasmids in all five genomes. Mauve alignment [24] of chromosomes of the five strains revealed a remarkably consistent backbone (Figure 4A-D). Compared with WSM1271, WSM2073, and WSM2075, synteny blocks of the 7653R chromosome shared a longer average length and more consistent relative positions with MAFF303099. Additionally, fewer sequence inversions were observed between the chromosomes of 7653R and MAFF303099 than between 7653R and WSM1271, WSM2073, or WSM2075. We used OrthoCluster [25, 26] to identify synteny blocks perfectly conserved between each pair of these five genomes, and assigned a score to each 7653R gene according to the length of the synteny (e.g., if gene A is in a synteny of seven genes, the score of gene A would be seven). We found that the mean score of all genes in synteny between 7653R and MAFF303099 was larger (10.46) than the mean scores between 7653R and WSM1271 (7.92), WSM2073 (8.24), or WSM2075 (8.86). Additionally, we performed a statistical test of significance, the results of which are shown Additional file 3: Table S3. Moreover, analyses of phylogenetic relationships based on the consistency of DNA sequences using Mauve and the Composition Vector using Cvtree [27] both indicated that 7653R has a closer relationship to MAFF303099 than to the other three *Mesorhizobium* strains (Figure 4E).

Thus, both ortholog and synteny analyses support a closer phylogenetic relationship between 7653R and MAFF303099 than with the other *Mesorhizobium* strains. These results provide further evidence that MAFF303099 is a strain of *M. huakuii*.

### Host specificity

Although 7653R and MAFF303099 are both strains of the same species, *M. huakuii*, they display drastically different host specificities. While the strain 7653R forms a specific symbiosis with *A. sinicus*, MAFF303099 forms symbioses with several *Lotus* species host plants, including *L. japonicus* and *L. corniculatus*[11, 16]. We aimed to determine what genomic features are responsible for such unique host preferences. Host specificity, an important trait underlying the interaction of rhizobia with their hosts, is still poorly understood [28]. Host switching or host jumping can often be traced to the modification of key microbial genes that facilitate the formation of particular host associations [29]. Because the determinants of host specificity of a bacterium mainly depend on three groups of signaling molecules—nodulation factors (NFs), surface polysaccharides, and secreted proteins [30], we explored genes that affect the biological synthesis of these signaling molecules in the genomes of these two strains and compared them with those of native *M. loti* strain R7A.

#### Nodulation factors

**Figure 5:**
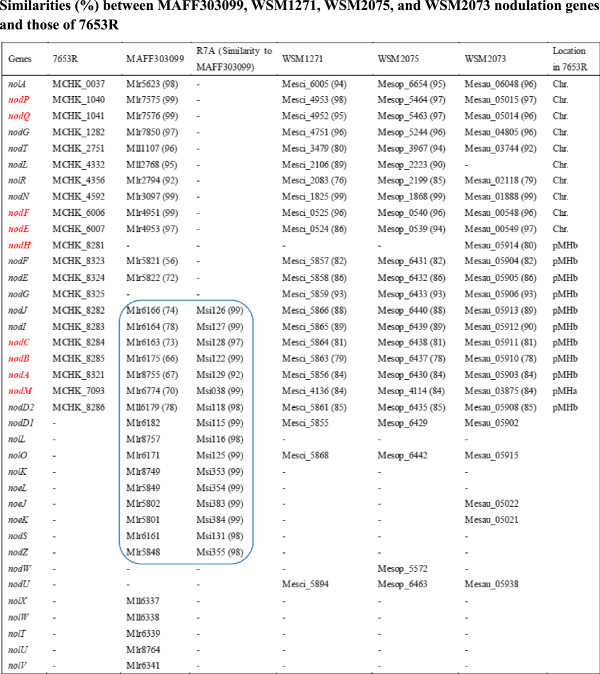
**Similarities (%) between MAFF303099, WSM1271, WSM2075, and WSM2073 nodulation genes and those of 7653R.**

NFs, which are signaling molecules between symbiotic bacteria and plants, are produced by bacteria in response to flavonoids secreted by legume root hairs [31]. In most rhizobia, expression of nodulation genes (*nod*, *nol*, and *noe*) is needed for the biosynthesis and transport of NFs that induce nodule organogenesis. A total of 21 nodulation genes (2 *nol* genes and 19 *nod* genes) were identified in the 7653R chromosome and plasmids, while 33 nodulation genes were located in MAFF303099 (Figure 5). In contrast, in R7A, 24 nodulation genes were found to be present and all were found to be homologous with very high similarities to genes in MAFF303099. Comparison of nodulation genes between these three strains not only revealed some genes with high sequence similarity but also uncovered two striking differences likely related to NF synthesis.

**Figure 6:**
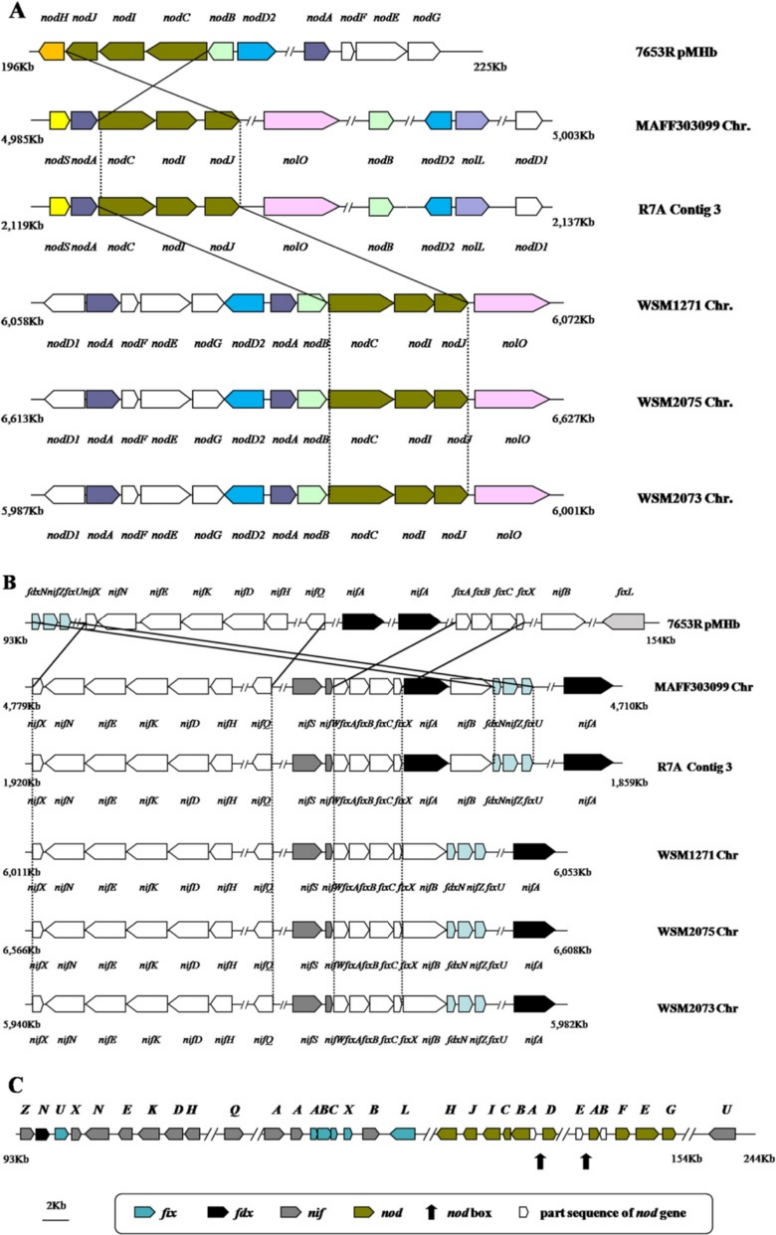
**Arrangement of nodulation genes (A), nitrogen fixation genes (B), and gene clusters in the 7653R plasmid pMhu7653Rb (C).** Double slash marks represent genome regions that are not shown. In the clusters, nitrogen-fixation genes **(B)** conserved among the six strains are represented by white arrows, while those varying in copy number, location, or transcriptional orientation are shown in different colors. Nodulation and nitrogen-fixation gene clusters **(C)** in 7653R plasmid pMhu7653Rb. Genes are colored according to their names. Double slash marks represent genome regions that are not shown. Arrows indicate the location of potential *nod*-boxes.

First, genomic distribution of these nodulation genes was different between 7653R and MAFF303099. While all 33 nodulation genes in MAFF303099 were found on its chromosome, only 10 nodulation genes were present on the chromosome of 7653R, with 11 found on its plasmids (1 on pMhu7653Ra and 10 on pMhu7653Rb). Specifically, the 10 *nod* genes (*nodA*, *B*, *C*, *D2*, *E*, *F*, *G*, *H*, *I*, and *J*) were identified in a 140-kb genomic region of the pMhu7653Rb plasmid of 7653R (Figure 6A). This genomic region also contained 6 *fix* genes (*fixA*, *B*, *C*, *L*, *U*, and *X*) and 12 *nif* genes (*nifB*, *D*, *E*, *H*, *K*, *N*, *Q*, *U*, *X, Z,* and two copies of *nifA*) (Figure 6B, C). The 10 *nod* genes were well conserved across all six genomes (Figure 5), as were the 6 *fix* genes and 12 *nif* genes (Additional file 1: Table S4). Although these *nod* genes individually exhibited strong conservation, major differences were observed in regard to their arrangement in the genomes. For example, the 10 *nod* genes on the pMhu7653Rb plasmid of 7653R were found to be segregated into two independent operons preceded by two *nod*-boxes (Figure 6A, C), with *nodA* separated from *nodBC* by a 22-kb genome region containing 35 genes [17]. In sharp contrast, orthologs of *nodA* and *nodBC* in other *Mesorhizobium* strains, including MAFF303099 and R7A [10], are adjacent and localized on the same strand (Figure 6A).

**Figure 7:**
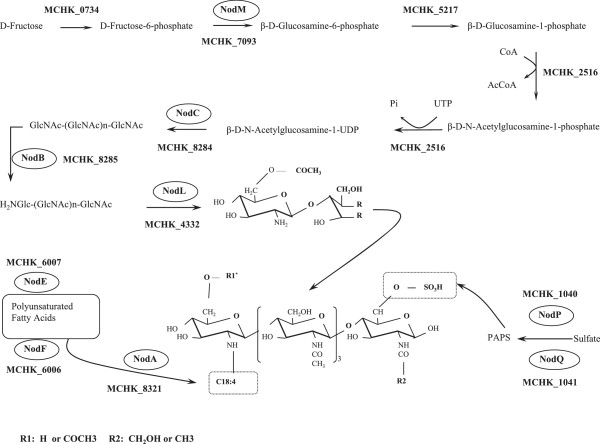
**Nodulation factor (NF) biosynthesis pathway in** ***M. huakuii*** **7653R.** Biosynthesis pathway of the core NFs of strain 7653R and involved Nod proteins are presented. Two variable ends of the chemical structure of the core NFs, R1 and R2, are shown in the figure. R1 thus far seems to be represented only by H in the 7653R NF structures.

Second, the numbers of nodulation genes putatively participating in NF synthesis were found to be different between these two strains. The *nod* gene *nodH,* required for NF synthesis in 7653R [32], had no ortholog in MAFF303099 and R7A (Figure 5). Each of the four *nod* genes in MAFF303099 (*nodM*, *C*, *B*, and *A*) had a substantially higher percentage identity (PID) with its ortholog in R7A than with its ortholog in 7653R (Figure 5). For example, *nodC* in MAFF303099 had a PID of 99% with its ortholog in R7A and a PID of 74% with its ortholog in 7653R (Figure 5). Interestingly, seven nodulation genes in MAFF303099 with orthologs in both 7653R and R7A were found to have substantially higher PIDs with their orthologs in R7A than with those in 7653R; nine nodulation genes in MAFF303099 had high PIDs with their orthologs in R7A but had no orthologs in 7653R. These results suggest that MAFF303099 may have obtained these 16 nodulation genes from an ancestor of R7A. Thus, although MAFF303099 shares 10 nodulation genes with high PIDs (>92%) with 7653R, it shares 24 nodulation genes with high PIDs with R7A. Furthermore, MAFF303099 was found to have an additional five nodulation genes (*nolT*, *U*, *V*, *W*, and *X*). Taken together, 7653R and MAFF303099 have very different numbers of nodulation genes. Indeed, different nodulation genes are required for NF synthesis in these two strains. Of the 21 *nod* genes identified in 7653R, 12 (*nodM, C, B, L, A, H, P, Q,* and two copies of *nodE and F*) are possibly key elements involved in the synthesis of the core NFs of 7653R [32] (Figure 7). In the *M. loti* strain R7A, *nod* genes organized in seven transcriptional units—*noeKJ*, *nodZnoeLnolK*, *nodS*, *nodACIJnolO*, *nodB*, *nolL,* and *nodM*—are needed for NF synthesis [12] (Additional file 4: Figure S1). Considering that many of the MAFF303099 nodulation genes showed higher PIDs with R7A genes, we further propose that nodulation genes required for the synthesis of NFs in MAFF303099 are closely related to those in R7A. This inference is consistent with a report that MAFF303099 and R7A may share the same steps of NF synthesis [32].

#### Surface polysaccharides

Rhizobial cell-surface polysaccharides, including cyclic-β-glucans (CβGs), exopolysaccharides (EPSs), lipopolysaccharides (LPSs), and capsular polysaccharides (KPSs or K-antigens), are necessary for establishing successful symbiosis with their hosts to form effective root nodules [33]. Comparative genomics analysis revealed that the genes needed for the biosynthesis of CβGs (*ndvA* and *ndvB*), EPSs (26 *exo*/*exs* genes; in Additional file 1: Table S5), and LPSs (Additional file 4: Figure S2 and Additional file 1: Table S6) are well conserved in all six genomes, suggesting that genes involved in the biosynthesis of surface polysaccharides are unlikely to contribute substantially to host preference differentiation.

#### Secretion system

Proteins secreted by some rhizobial strains play an important role in infection of leguminous plant roots and establishment of a mutually beneficial symbiosis. Different types and numbers of protein secretion systems are present in almost all rhizobial strains. By means of similarity searches using protein secretion genes identified in other Gram-negative bacteria as queries, we identified 101 genes related to secretory processes in the 7653R genome. These genes and proteins are involved in 12 putative protein secretion systems: a general export pathway, four separate type-I systems, a twin-arginine translocase secretion system, one functional type-III system (T3SS), two type-IV systems (T4SSs), one type-V autotransporter, and two putative type-VI secretion systems (T6SSs) (Table 2).

**Table 2:** **Numbers and distributions of genes associated with different types of secretion systems in mesorhizobial genomes**

Secretion system and characteristics	Gene numbers associated with the formation of different types of secretion systems					
	7653R	MAFF303099	R7A	WSM1271	WSM2075	WSM2073
**Type I**						
AprD	4	3	2	2	2	3
AprE	4	3	1	2	2	3
TolC	2	1	1	2	2	2
**Type II**						
GSP (general secretion pathway)		11	8	11		
Sec pathway	7	7	7	7	7	7
**Type III**						
Rhc pili	22	22				
**Type IV**						
F-type (Vir)	10	10	9	12	12	11
F-type (Trb)		19	10	7	9	16
P-type (Flp and attachment)	16	16	15	16	16	16
**Type V**						
Autotransporter	1	1	1	1	1	1
**Type VI**	26	18	18	18		
**TAT** (twin arginine)	3	3	3	3	3	3
**SRP**	5	5	5	5	5	5
**Mechanosensitive channels**	1	1	1	1	1	1
**Total**	101	120	81	87	60	68

Our comparative analysis of these secretion systems in the genomes of the two *M. huakuii* strains revealed important differences in three secretion systems: T3SS, T4SS, and T6SS. Gene clusters encoding the major and conserved components of T3SSs are present in diverse and distantly related rhizobia [34, 35]. The 7653R genome was found to contain a complete T3SS on the pMhu7653Rb plasmid, with gene organization conserved with respect to MAFF303099. Proteins secreted by rhizobial T3SS are called nodulation outer proteins (Nops) and can be divided into two types: effectors and helper proteins. T3SSs of both 7653R and MAFF303099 have three helper proteins, NopA, NopB, and NopX, but different candidate effectors: NopP in 7653R and NopC in MAFF303099 (Additional file 5: Table S7). Although T3SS and its secreted effectors are dispensable for rhizobial infection and nodulation, they may function as facilitators superimposed on the Nod-factor signaling pathway and modulate host range in a genotype-specific manner [28]. Thus, T3SS might be one determinant of host range variation in 7653R and MAFF303099. The Vir system, an important example of a T4SS, is usually formed by 12 proteins, VirB1–VirB11 and VirD4. Except for VirB1 and VirB7, these proteins are encoded by genes on plasmid pMhu7653Ra. Interestingly, neither VirB1 nor VirB7 are present in MAFF303099 and R7A [14]. The Vir systems of 7653R and MAFF303099 are thus essentially identical. In contrast, the T4SS Trb system was found to differ between 7653R and the other five *Mesorhizobium* strains; in particular, 7653R has no *trb* gene, whereas MAFF303099 has 19 *trb* genes (Table 2). The T6SS apparatus is assembled by a conserved set of proteins whose functions are closely related to bacterial pathogenesis and host cell survival [36]. Two T6SSs were found in the 7653R genome, while one each was identified in MAFF303099 and R7A genomes (Table 2).

Taken together, our analysis revealed that the two *M. huakuii* strains 7653R and MAFF303099 have substantial differences in the number and arrangement of genes responsible for synthesizing NFs, and also differ with respect to secretion systems T3SS, T4SS, and T6SS. These differences may contribute to the establishment of differential host specificity.

Changes in host specificity determinants—for example, by acquisition of new genetic elements that grant a selective advantage in a particular host environment—can have a great impact on host range and may lead to host jumps [29]. Both intrageneric and intergeneric HGT have been reported as important mechanisms for the spread of symbiotic capacity in the Salado River Basin [16]. Intrageneric HGT might be a main pathway to change symbiotic capacity in MAFF303099. *Mesorhizobium* strains isolated from *A. sinicus* in Japan, designated as *M. huakuii* subsp. *rengei,* are able to coexist with *M. loti* strains and thus have a chance to exchange genetic information through conjugation. The ancestral strain of *M. huakuii* presumably derived some genetic information from native *M. loti* strains, thereby introducing genetic variation in host specificity determination. The ancestral strain eventually evolved into strain MAFF303099, which can form an effective symbiotic relationship with *Lotus corniculatus*. The introduction of novel genetic variation by HGT is typically accompanied by the acquisition and incorporation of genetic fragments or intact transcriptional units into the genome [37]. Although NFs and secreted effectors of T3SS in MAFF303099 are associated with genetic fragments and intact transcriptional units, we still cannot confirm the underlying causes of the host specificity changes: there may be a continuum that ranges from changes in single residues to gene domains, whole genes, and eventually entire genomic islands (GEIs) [29]. Consequently, much remains to be learned about whether many or only a few gene loci are involved in the determination of nodulation specificity. Moreover, genes from leguminous plants, such as the *R*-gene from soybean [28], can also participate in the control of genotype-specific infection and nodulation.

### Symbiosis island dynamics and the origin of symbiotic plasmids

**Figure 8:**
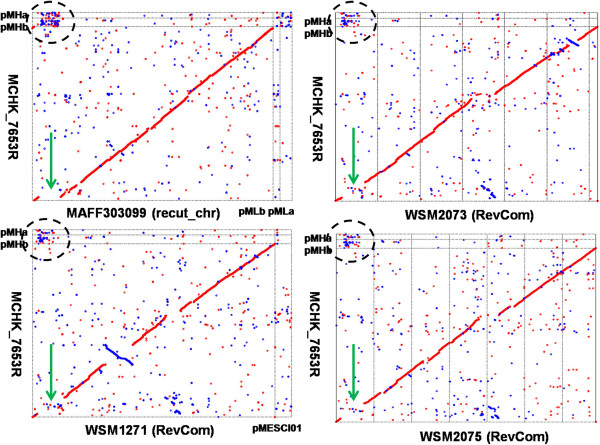
**Four mesorhizobial genomes plotted against the 7653R genome using PROmer (PROtein MUMmer).** A sequence aligned according to the MAFF303099 replication origin and reverse-complement sequences of WSM2073, WSM1271, and WSM2075 were used. Line figures depict the results of PROmer analysis. Red dots represent similar sequences in the forward direction in each genome pair, whereas blue dots indicate that the similarity is in the opposite orientation. Green arrows show the location of fragments missing on the 7653R chromosome.

Although the chromosomes of 7653R and MAFF303099 showed good overall co-linearity, a large, approximately 610-kb genomic region unique to MAFF303099 was identified (Figure 4A). Comparison of 7653R genomic structures to genomes of R7A and MAFF303099 using the ACT (Artemis comparison tool) [38] confirmed this observation (Additional file 4: Figure S3). Such genome-specific sequences were also noticed in similar positions in the other three genomes (WSM1271, WSM2075, and WSM2073) (Figure 4B–D), which was verified through genome alignment using PROmer (PROtein MUMmer) [39] (Figure 8). These genome-specific regions harbor most nodulation and nitrogen-fixation genes. Interestingly, homologs of these nodulation and nitrogen-fixation genes can be traced to the two 7653R plasmids, suggesting a connection between the ‘missing’ DNA fragment and these symbiotic plasmids (Figure 8).

Of the five *Mesorhizobium* strains whose genomes have been completely sequenced (excluding R7A with its incomplete genome data), only 7653R has symbiotic plasmids. In contrast, all other strains either have no plasmids, or their plasmids do not contain genes involved in symbiosis. Thus, while the nodulation and nitrogen-fixation genes are localized on the plasmids as a symbiosis island in 7653R, they are localized on the main chromosomes of the other four strains. Global genome alignment between 7653R and the other genomes revealed that the symbiosis islands are positioned in a synteny gap region that corresponds to the genome-specific region in MAFF303099 and the gap in 7653R (Figure 8 and Additional file 4: Figure S3), suggesting that the plasmids were excised from the main 7653R chromosome. Plasmids of 7653R and these genome-specific regions found in the other four genomes are thus likely GEIs. To test this hypothesis, we examined these genome-specific regions i.e., symbiosis islands, using IslandViewer, a program for finding GEIs [40]. As expected, IslandViewer identified these MAFF303099, WSM1271, WSM2073, and WSM2075 symbiosis islands as typical GEIs (Additional file 4: Figure S4). These predictions are supported by the results of further analysis of genomic features. First, plasmids of 7653R and the other four GEIs have similar sizes (514–611 kb) and similar GC content (58–59%), which is strikingly lower than that of the corresponding genome (62.51–62.87%). Second, codon usage of 7653R plasmid ORFs is significantly different from that of the chromosome but surprisingly consistent with those of the other four GEIs (Additional file 4: Figure S5). Third, T3SSs and/or T4SSs of the five strains are all located in the corresponding candidate GEIs. Fourth, a highly conserved tRNA(Gly) gene is found in the vicinity of the candidate GEI in all five *Mesorhizobium* strains except for 7653R. In 7653R, plasmids possess the same characteristics as the other four GEIs located in specific genome regions. We propose that the plasmids of 7653R were formed during evolution by the excision of the GEI from the 7653R chromosome, as described previously in other systems [41].

Because the five GEIs likely share a common ancestor, we expected them to maintain well-conserved syntenic relationships. Although the GEIs in WSM1271, WSM2075, and WSM2073 displayed conserved synteny, the GEIs in these three strains and two other strains surprisingly showed little resemblance in regard to gene organization. We noticed that 80% of all transposase genes in the entire 7653R genome are concentrated on its plasmids. This enrichment of transposase genes on the plasmids of 7653R resembles that of the MAFF303099 GEI, which possesses 89 predicted transposase genes—86% of all transposase genes in the entire MAFF303099 genome. Similarly, 85% (41) of all transposase genes identified in the entire contigs of R7A are found in the symbiosis island of contig 3. In contrast, the GEIs of the other three *Mesorhizobium* strains harbor only a few transposase genes, and they show highly conserved synteny. On the basis of this observation, we propose that the enrichment of transposase genes in the GEIs of 7653R and MAFF303099 caused a disruption in gene order within their GEIs, whereas the lack of transposase genes in the other three *Mesorhizobium* strains helped to maintain their GEI synteny. The question then arises: what is the source of these transposase genes in the GEIs of 7653R and MAFF303099? One likely source is HGT. Previous analysis of nodulation genes has proved that the GEI of MAFF303099 has acquired many foreign genes by HGT [42]. Our clustering analysis of transposase genes in the plasmids of 7653R and the MAFF303099 GEI revealed that most of them belong to different families, suggesting that these transposase genes were likely acquired via HGT. Thus, these five *Mesorhizobium* strains may have inherited their GEIs from a common ancestral GEI, which later underwent various degrees of change.

**Figure 9:**
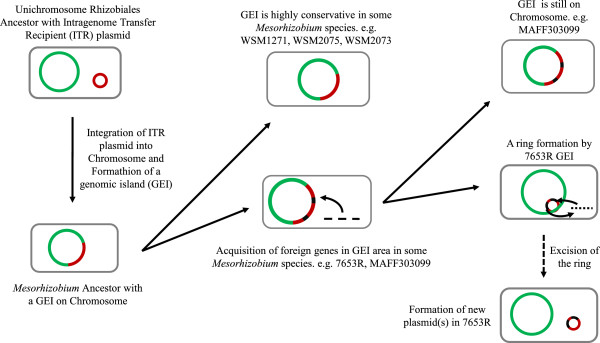
**The contribution of genome dynamics to general** ***Mesorhizobium*** **evolution.** Chromosomal, ITR plasmid, and foreign genes are shown in different colors.

It has been speculated that GEIs may be derived from integrating mobile genetic elements such as plasmids or phages. Their acquisition by HGT and integration with the host chromosome by site-specific recombination might lead to the formation of a new GEI [37]. Slater et al. have proposed that integration of an ancestral intragenomic translocation recipient (ITR) plasmid into the main chromosome is an important evolutionary pathway in *Rhizobiales*[43]. *Bradyrhizobium* and *Mesorhizobium* strains with a few or no relatively small plasmids are typically cited as examples, although the sole evidence for this viewpoint is the presence of ITR plasmid gene clusters and other plasmid genes on the chromosomes of these species. Aside from plasmid genes shown on chromosomes, further evidence based on genomic structure, nucleotide composition, and transposase genes was used in this study to infer a possible evolutionary pathway explaining GEI formation (Figure 9). In our proposed scenario, integration of an ITR plasmid into an ancestral *Mesorhizobium* main chromosome would be followed by the formation of a new GEI—the original parent of the present-day chromosomal GEIs of the four fully sequenced *Mesorhizobium* strains. Because the evolving strains lived under different environmental conditions and experienced different selection pressures, the new GEIs underwent various changes at different rates. GEIs of some strains, such as WSM1271, WSM2075, and WSM2073, maintained high conservation under weak selection pressures. GEIs of strains such as 7653R and MAFF303099, however, underwent frequent recombination events that created high levels of instability. In particular, GEIs of 7653R and MAFF303099 both encode mobility enzymes, such as integrase, that allow excision from the host chromosome. Nevertheless, only the original GEI of 7653R can excise itself spontaneously from the chromosome and form replicable plasmids. The GEI of MAFF303099 may have become immobilized because of failure to regain the origins of plasmid replication or the genes involved in mobilization [37].

Many transposase genes exist within GEIs of 7653R and MAFF303099. Except for several conserved but inactive genes, these genes were acquired from foreign species. The transposases encoded by foreign genes have retained high activity, indicating a continuous exchange of 7653R and MAFF303099 genetic information with other species. How rhizobial genomes are able to select the proper foreign genes while still maintaining structural stability and gene function despite the disruption remains unknown. Complex cellular programs associated with some bacterial traits, such as symbiosis, must exist to ensure adaption to the surrounding environment and to maintain competitiveness. A large body of research has confirmed this point. In one recent case, genes on a genomic island were reported to confer an adaptive advantage to specific stresses in marine *Synechococcus*[44]. For better survival and growth in various habitats, GEIs from MAFF303099 acquired some foreign nodulation genes by HGT during the genetic information exchange process, enabling functional symbiosis between MAFF303099 and a new host plant. Furthermore, the acquisition of foreign genetic elements is frequently accompanied by the loss of native genes. As to the argument that the lost genes are randomly selected or under special selection, increasing evidence inclines to the view that loss of functionality can be a selective advantage in some specific situations [45].

In *Legionella pneumophila,* a newly identified conjugation/type-IVA secretion system (trb/tra) composed of clusters of *tra* and *trb* genes (related to the Vir system and conjugal transformation) seems to be necessary for integrase-dependent excision and horizontal transfer of GEIs [46]. A similar system has been identified on the other four GEIs, excluding 7653R, with different sets of *tra* and *trb* genes scattered on them. The existence of the same set of *tra* and *trb* genes with high similarities in strains MAFF303099, WSM1271, WSM2075, WSM2073, and R7A [12] indicates that the ancestral ITR plasmid that integrated into chromosomes of ancestral *Mesorhizobium* strains contained a functional conjugation/type-IVA secretion system. Plasmid pMhu7653Ra of 7653R, however, has only a few *tra* genes and no *trb* gene. Integrated mobile elements should theoretically be inactivated or lose genes related to plasmid mobilization or transfer, such as *tra* and *trb*. It is difficult to judge whether the IVA systems are inactive or if some of the key *tra*-*trb* genes have already been deleted from the GEIs of MAFF303099, WSM1271, WSM2075, and WSM2073. To determine what happened to the *tra*-*trb* genes on the GEI of 7653R chromosome before excision, further bioinformatics analysis and experimental evidence are needed.

## Conclusions

Whole-genome sequencing has proven valuable and critical for refining the phylogenetic positions of a series of rhizobial strains [47]. In this study, we sequenced, assembled, and annotated the *M. huakuaii* 7653R genome. We used this genome sequence to examine the phylogenetic position of MAFF303099, a strain whose phylogenetic position has been debated. These two strains share a large set of orthologs and, most importantly, a conserved chromosomal backbone and even larger perfectly conserved synteny blocks. Our ortholog and synteny analyses have firmly placed MAFF303099 as a strain of *M. huakuii*, as is 7653R.

Although 7653R and MAFF303099 are both strains of *M. huakuii*, they exhibit important differences in symbiotic phenotypes and thus belong to different symbiosis variants (also known as symbiovars) [48]. This placement is supported by our analysis of nodulation and fixation genes, which revealed notable differences in several nodulation genes, mostly related to NF generation. Such differences have a profound impact on host specificity. In a few rhizobium strains, mutations of some specific genes related to NFs and T3SS have been found to alter host specificity; additionally, the distribution of nodulation genes is reportedly related to requirements for effective symbiosis with some legume hosts [49–51]. Furthermore, our analysis of the three groups of signaling molecules revealed substantial differences between the two *M. huakuii* strains 7653R and MAFF303099 that were focused on the number and arrangement of genes responsible for synthesizing NFs and secretion systems T3SS, T4SS, and T6SS. In conjunction with NFs, these secretion systems may contribute to the establishment of differential host specificity.

Our results strongly suggest a common site-specific GEI localization mechanism in the ancestral *Mesorhizobium* chromosome, with the GEIs of the genus showing different degrees of variability after divergence from the mesorhizobial ancestor. A similar phenomenon has been observed in *Bradyrhizobium japonicum* strains. Various lines of evidence support past horizontal insertion of GEIs into the ancestral genome of *B. japonicum* USDA110, and comparative genomic hybridization profiles show that GEIs may be highly dynamic entities in *B. japonicum* genomes [52]. The ability of integrating mobile genetic elements to enlarge chromosomes may be due to the fact that *Bradyrhizobium* and *Mesorhizobium* species have very large chromosomes with few plasmids [43]. The recent completion of genome-sequencing projects for several *Mesorhizobium* species has enabled analysis of the global changes between them after the acquisition and integration of the ancestral ITR plasmid. An improved understanding of these variations should improve our understanding of how genome dynamics can contribute to bacterial evolution in general.

7653R plasmids possess the same characteristics as the GEIs of the other four *Mesorhizobium* genomes. Additionally, homologs of nodulation and nitrogen-fixation genes on the other four GEIs are found on the two plasmids of 7653R. Moreover, it has been reported that GEIs can excise themselves spontaneously from the chromosome and form plasmids with the acquisition of functions for autonomous replication (e.g., *repABC* genes) or can be transferred to other suitable recipients [53]. We therefore conclude that 7653R plasmids may have arisen by the excision of the original GEI from the 7653R chromosome.

## Methods

### Bacterial strains and DNA preparation

*Mesorhizobium huakuii* 7653R was cultured for 3 days at 28°C in trypticase-yeast extract medium. Cells of 7653R were harvested by centrifugation, with total DNA prepared using a Genomic DNA Mini Preparation kit.

### Sequencing and annotation

For *de novo* sequencing of the 7653R genome, a combined strategy comprising Solexa sequencing on an Illumina GAIIx platform was carried out by BGI (Beijing Genomics Institute, Beijing, China). As a result, 367 contigs were generated with a 29-fold median coverage depth.

Sequence assembly was performed using SOAPdenovo [54], with PCR-based amplicon sequencing used for gap closure. Glimmer 3.0 [55], RNAmmer 1.2 [56], and tRNAscan-SE [57] were used respectively for *de novo* prediction of genes, rRNA genes, and tRNAs. Clusters of Orthologous Groups (COG) annotation was performed using RPS-BLAST against the CDD database [58], and Gene Ontology annotation was carried out with InterProScan V4 [59]. A bidirectional best hit approach (*E*-value < 1 × 10^−5^, identity > 30%, coverage > 70%, and bit score > 60) was used for KEGG [60] and SWISS-PROT [61] annotations.

### Genome comparisons

The complete nucleotide sequences of strains MAFF303099, WSM1271, WSM2075, and WSM2073 were obtained from GenBank (accession numbers: *M. huakuii* bv. *loti*, NC_002678, NC_002679, and NC_002682; *M. ciceri*, NC_014923 and NC_014918; *M. opportunistum*, NC_015675; *M. australicum*, NC_019973). The sequences were organized according to their chromosomal origins of replication for intuitive comparison. Sequences of three contigs from R7A were obtained from the JGI Genome Portal (Project ID: 404030). Genome sequence alignments were created using MUMmer, ACT, and Mauve software.

### Ortholog analysis

The OrthoMCL [62] approach was adopted to construct gene families for all coding sequences in the five *Mesorhizobium* genomes. Quartets of orthologous proteins (quartops) in all pairwise genome comparisons were considered to constitute the ‘core’ genome. Proteins with no homologs in the other four *Mesorhizobium* genomes were defined as differential genes.

### Nucleotide sequence accession numbers

Complete genome sequences of *M. huakuii* 7653R have been submitted to GenBank under the following assigned accession numbers: *Mesorhizobium* CP006581; *Mesorhizobium*_1 CP006582; *Mesorhizobium*_2 CP006583.

## Electronic supplementary material

Additional file 1: Tables S1, S4, S5, and S6: **Table S1** The numbers and types of tRNAs in five mesorhizobial genome. **Table S4** Similarities (%) for nitrogen fixation genes of 7653R, R7A, WSM1271, and WSM2075 in comparison with those of MAFF303099. **Table S5** Similarities (%) of EPS biosynthesis genes of 7653R, WSM1271, WSM2075, and WSM2073 in comparison with those of MAFF303099. **Table S6** Similarities (%) of LPS biosynthesis genes of 7653R in comparison with those of MAFF303099, WSM1271, WSM2075, and WSM2073. (PDF 187 KB)

Additional file 2: Table S2: A list of 210 conserved genes in *Mesorhizobium huakuii* 7653R used for hierarchical clustering analysis. (XLSX 148 KB)

Additional file 3: Table S3: *P*-values for *t*-test of significance. (XLSX 9 KB)

Additional file 4: Figures S1 to S5: **Figure S1** Nodulation genes participating in NF synthesis. *nodE* and/or *nodF* from the 7653R chromosome, pMhu7653Rb, or both participate in synthesis of NFs. **Figure S2** Lipopolysaccharide biosynthesis pathway in *Mesorhizobium huakuii* 7653R. Biosynthesis substrates and products and key enzymes of each step are indicated. **Figure S3** ACT visualization of 7653R, R7A, and MAFF303099 chromosomes and plasmids. Genomic alignment of strains 7653R, R7A, and MAFF303099 was performed using ACT [38]. Red connections represent syntenic regions; blue ones represent inversions. The R7A genome with contigs in the order of contigs 1, 2, and 3 is at the top of the figure. The 7653R genome with replicons in the order of Chromosome, pMhu7653Ra and pMhu7653Rb is in the middle and the MAFF303099 genome is at the bottom in the order of Chromosome, pMLa, and pMLb. **Figure S4** Genomic islands (GEIs) predicted for the four *Mesorhizobium* strains by IslandViewer. GEIs are shown for MAFF303099 (A); WSM1271 (B); WSM2073 (C); and WSM2075 (D). Genomes in EMBL or GENBANK format are used. The green ellipse indicates the position of the GEI, which is the same as the symbiosis island on each chromosome. **Figure S5** Comparison of codon usage among genomic islands (GEIs) in *Mesorhizobium*. Codon usage patterns were compared between GEIs and the remaining chromosomes. Lysine codon usages are not included because of the huge variability. (PDF 1 MB)

Additional file 5: Table S7: Proteins related to the type-III secretion system in *Mesorhizobium huakuii* 7653R and MAFF303099. (XLSX 138 KB)

## Abbreviations

CβG: Cyclic-β-glucan
EPS: Exopolysaccharide
GEI: Genomic island
HGT: Horizontal gene transfer
ITR: Intragenomic translocation recipient
KPS: Capsular polysaccharide
LPS: Lipopolysaccharide
PID: Percentage identity
NF: Nodulation factor.
