# Supplementary material for: Whole-genome sequencing of Mesorhizobium huakuii 7653R provides molecular insights into host specificity and symbiosis island dynamics
Source: BMC Genomics. 2014 Jun 6;15(1):440. doi: 10.1186/1471-2164-15-440 (PMC4072884; doi:10.1186/1471-2164-15-440)
Supplement: Supplementary file 1 — Additional file 1: Tables S1, S4, S5, and S6: Table S1 The numbers and types of tRNAs in five mesorhizobial genome. Table S4 Similarities (%) for nitrogen fixation genes of 7653R, R7A, WSM1271, and WSM2075 in comparison with those of MAFF303099. Table S5 Similarities (%) of EPS biosynthesis genes of 7653R, WSM1271, WSM2075, and WSM2073 in comparison with those of MAFF303099. Table S6 Similarities (%) of LPS biosynthesis genes of 7653R in comparison with those of MAFF303099, WSM1271, WSM2075, and WSM2073. (PDF 187 KB) [file 12864_2013_6156_MOESM1_ESM.pdf]

Additional file 1

This file contains 4 supplemental tables, Tables S1, S4, S5, S6.

Table S1 The numbers and types of tRNAs in five mesorhizobial genome

	7653R	MAFF303099	WSM1271	WSM2075	WSM2073
**rRNA**					
5s rRNA	1	2	2	2	2
16s rRNA	2	2	2	2	2
23s rRNA	2	2	2	2	2
Total	5	6	6	6	6
**tRNA**					
Ala	5	4	4	4	4
Gly	3	3	3	3	3
Pro	3	3	4	4	4
Thr	3	3	3	3	3
Val	3	3	3	3	3
Ser	4	4	4	4	4
Arg	4	4	4	5	4
Leu	5	5	5	5	5
Phe	1	1	1	1	1
Asn	2	1	2	1	1
Lys	2	2	2	2	2
Asp	1	1	1	1	1
Glu	2	2	2	2	2
His	1	1	1	1	1
Gln	3	3	3	3	3
Ile	2	3	2	2	2
Met	3	4	4	5	5
Tyr	1	1	2	1	2
Cys	2	1	2	2	2
Trp	1	1	1	1	1
Total	51	50	53	53	53

Table S4 Similarities (%) for nitrogen fixation genes of 7653R, R7A, WSM1271, and WSM2075 in comparison with those of MAFF303099

Genes	MAFF303099	7653R	R7A	WSM1271	WSM2075	WSM2073	Location in 7653R
FixS	Msl6623	MCHK_0351 (82)	Msi010 (99)	-	-	Mesau_02498 (78)	Chr.
	Msr6418						
FixI	Mll6624	MCHK_0352 (82)	Msi011 (90)	Mesci_6043 (89)	Mesop_6032 (91)	Mesau_05552 (90)	Chr.
	Mlr6417			Mesci_5504 (90)	Mesop_6085 (90)	Mesau_02499 (81)	
FixH	Mll6625	MCHK_0353 (87)	Msi012 (84)	Mesci_5505 (88)	Mesop_6086 (88)	Mesau_05553 (88)	Chr.
	Mlr6416			Mesci_6044 (83)	Mesop_6031 (83)	Mesau_02500 (84)	
FixG	Mll6626	MCHK_0354 (96)	Msi013 (87)	Mesci_6045 (93)	Mesop_6030 (92)	Mesau_02501 (91)	Chr.
	Mlr6415			Mesci_5506 (86)	Mesop_6087 (86)	Mesau_05554 (87)	
FixP	Mll6628	MCHK_0355 (90)	Msi014 (88)	Mesci_6046 (86)	Mesop_6029 (87)	Mesau_02502 (87)	Chr.
	Mlr6414			Mesci_5507 (80)	Mesop_6088 (80)	Mesau_05555 (82)	
FixQ	Msl6627	MCHK_0356 (99)	Msi015 (88)	Mesci_6047 (82)	Mesop_6028 (76)	Mesau_02503 (84)	Chr.
	Msr6413			Mesci_5508 (67)	Mesop_6089 (67)	Mesau_05556 (68)	
FixO	Mll6629	MCHK_0357 (97)	Msi016 (93)	Mesci_6048 (94)	Mesop_6027 (94)	Mesau_02504 (95)	Chr.
	Mlr6412			Mesci_5509 (94)	Mesop_6090 (94)	Mesau_05557 (94)	
FixN	Mll6630	MCHK_0358 (99)	Msi017 (92)	Mesci_6049 (95)	Mesop_6026 (96)	Mesau_02505 (96)	Chr.
	Mlr6411			Mesci_5510 (90)	Mesop_6091 (90)	Mesau_05558 (90)	
FixJ	Mll6606	MCHK_3913 (83)	-	Mesci_6462 (91)	Mesop_6045 (84)	Mesau_02493 (84)	Chr.
FixK	Mll6578	MCHK_3897 (80)	-	Mesci_6459 (83)	Mesop_6050 (79)	Mesau_02489 (80)	Chr.
		MCHK_3951					
FixL	Mll6607	MCHK_3912 (83)	-	Mesci_6463 (94)	Mesop_6044 (85)	Mesau_02494 (87)	Chr.
		MCHK_8231					pMHb
FixU	Msl5852	MCHK_8164 (84)	Msi350 (96)	Mesci_5843 (75)	Mesop_6417 (75)	Mesau_05890 (75)	pMHb
FixA	Mll5862	MCHK_8217 (84)	Msi342 (98)	Mesci_5836 (84)	Mesop_6410 (84)	Mesau_05883 (85)	pMHb
FixB	Mll5861	MCHK_8218 (89)	Msi343 (99)	Mesci_5837 (86)	Mesop_6411 (86)	Mesau_05884 (86)	pMHb
FixC	Mll5860	MCHK_8219 (87)	Msi344 (99)	Mesci_5838 (86)	Mesop_6412 (86)	Mesau_05885 (86)	pMHb
FixX	Msl5859	MCHK_8220 (86)	Msi345 (99)	Mesci_5839 (86)	Mesop_6413 (86)	Mesau_05886 (86)	pMHb
NifV	Mlr7805	MCHK_1235 (99)	-	Mesci_4792 (94)	Mesop_5287 (96)	Mesau_04847 (96)	Chr.
NifS	Mlr0015	MCHK_1835 (96)		Mesci_4390 (91)	Mesop_4834 (93)	Mesau_04450 (86)	Chr.
	Mll5865		Msi340 (98)	Mesci_5833 (75)	Mesop_6407 (75)	Mesau_05880 (76)	
NifU	Mll0920	MCHK_2657 (97)	-	Mesci_4207 (95)	Mesop_4044 (94)	Mesau_03810 (95)	Chr.
FdxN	Msl8750	MCHK_8161 (80)	Msi348 (98)	Mesci_5841 (88)	Mesop_6415 (88)	Mesau_05888 (89)	pMHb
NifZ	Mll5854	MCHK_8163 (77)	Msi349 (97)	Mesci_5842 (76)	Mesop_6416 (76)	Mesau_05889 (77)	pMHb
NifN	Mlr5909	MCHK_8171 (82)	Msi290 (98)	Mesci_5813 (77)	Mesop_6389 (77)	Mesau_05862 (76)	pMHb
NifE	Mlr5908	MCHK_8172 (89)	Msi291 (95)	Mesci_5814 (89)	Mesop_6390 (89)	Mesau_05863 (89)	pMHb
NifK	Mlr5907	MCHK_8174 (92)	Msi292 (98)	Mesci_5815 (91)	Mesop_6391 (91)	Mesau_05864 (91)	pMHb
NifD	Mlr5906	MCHK_8175 (90)	Msi293 (99)	Mesci_5816 (92)	Mesop_6392 (92)	Mesau_05865 (92)	pMHb
NifH	Mlr5905	MCHK_8176 (96)	Msi294 (99)	Mesci_5817 (96)	Mesop_6393 (96)	Mesau_05866 (96)	pMHb
NifQ	Mlr5871	MCHK_8188 (70)	Msi336 (99)	Mesci_5823 (65)	Mesop_6398 (65)	Mesau_05872 (65)	pMHb
NifA	Mll5837	MCHK_8211 (67)	Msi361 (98)	Mesci_5851 (60)	Mesop_6425 (60)	Mesau_05898 (60)	pMHb
	Mll5857	MCHK_8214 (55)	Msi346 (96)				pMHb
NifB	Mll5855	MCHK_8226 (82)	Msi347 (99)	Mesci_5840 (79)	Mesop_6414 (79)	Mesau_05887 (79)	pMHb
NifX	Mlr5911	MCHK_8169 (79)	Msi289 (98)	Mesci_5812 (78)	Mesop_6388 (78)	Mesau_05861 (78)	
NifW	Mll5864	-	Msi341 (98)	Mesci_5835 (75)	Mesop_6409 (75)	Mesau_05882 (74)	

Table S5 Similarities (%) of EPS biosynthesis genes of 7653R, WSM1271, WSM2075, and WSM2073 in comparison with those of MAFF303099

Genes	MAFF303099	7653R	WSM1271	WSM2075	WSM2073
ExoB	Mlr5697	MCHK_0099 (98)	Mesci_5951 (96)	Mesop_6604 (97)	Mesau_05976 (96)
	Mll7878	MCHK_1305 (97)	Mesci_4728 (87)	Mesop_5223 (90)	Mesau_04782 (89)
ExoM	Mll6500	MCHK_0188 (95)	Mesci_2394 (91)	Mesop_2530 (92)	Mesau_02422 (87)
	Mlr5273	MCHK_6326 (97)	Mesci_0229 (84)	Mesop_0232 (87)	Mesau_00233 (86)
ExoZ	Mlr8032	MCHK_1438 (90)	-	-	-
ExoI	Mll8119	MCHK_1463 (91)	-	-	-
	Mll0560	MCHK_2272 (98)	Mesci_3932 (90)	Mesop_4449 (93)	Mesau_04087 (93)
	Mlr0479	MCHK_4012 (70)	Mesci_4973 (64)	-	-
ExoF	Mll5251	MCHK_6303 (99)	Mesci_0250 (87)	Mesop_0253 (95)	Mesau_00254 (93)
ExoP	Mlr6506	MCHK_0193 (84)	Mesci_2399 (74)	Mesop_2535 (84)	Mesau_02427 (69)
	Mlr8439	MCHK_1756 (99)	Mesci_4448 (81)	Mesop_4896 (84)	Mesau_04501 (87)
	Mlr5276	MCHK_6329 (97)	Mesci_0226 (90)	Mesop_0229 (93)	Mesau_00230 (95)
ExoR	Mll1100	MCHK_2741 (99)	Mesci_3484 (94)	Mesop_3973 (96)	Mesau_03749 (97)
ExoN	Mll3025	MCHK_4539 (99)	Mesci_1881 (96)	Mesop_1928 (97)	Mesau_01940 (96)
	Mlr5275	MCHK_6328 (99)	Mesci_0227 (99)	Mesop_0230 (99)	Mesau_00231 (95)
ExoS	Mll5094	MCHK_6150 (99)	Mesci_0468 (97)	Mesop_0396 (98)	Mesau_00386 (98)
ExoQ	Mlr5249	MCHK_6299 (99)	Mesci_0251 (88)	Mesop_0254 (93)	Mesau_00255 (91)
	Mll6499	MCHK_0187 (97)	Mesci_2393 (93)	Mesop_2529 (94)	Mesau_02421 (94)
ExoY	Mll5252	MCHK_6304 (84)	Mesci_0249 (95)	Mesop_0252 (96)	Mesau_00253 (96)
ExoX	Mlr5253	MCHK_6305 (99)	Mesci_0248 (78)	Mesop_0251 (79)	Mesau_00252 (86)
ExoU	Mlr5261	MCHK_6315 (98)	Mesci_0240 (95)	Mesop_0243 (95)	Mesau_00244 (95)
ExoK	Mlr5264	MCHK_6317 (98)	Mesci_0238 (85)	Mesop_0241 (86)	Mesau_00242 (87)
ExoT	Mll5270	MCHK_6323 (95)	Mesci_0232 (95)	Mesop_0235 (95)	Mesau_00236 (95)
ExoL	Mlr5271	MCHK_6324 (95)	Mesci_0231 (84)	Mesop_0234 (90)	Mesau_00235 (88)
ExoA	Mlr5272	MCHK_6325 (98)	Mesci_0230 (91)	Mesop_0233 (92)	Mesau_00234 (87)
ExoO	Mlr5274	MCHK_6327 (94)	Mesci_0228 (92)	Mesop_0231 (91)	Mesau_00232 (91)
ExpG	Mlr8394	MCHK_1710 (99)	-	Mesop_4936 (99)	Mesau_04537 (98)

Table S6 Similarities (%) of LPS biosynthesis genes of 7653R in comparison with those of MAFF303099, WSM1271, WSM2075, and WSM2073

Genes	7653R	MAFF303099	WSM1271	WSM2075	WSM2073
LpxA	MCHK_2336	Mll0633 (99)	Mesci_3972 (91)	Mesop_4265 (97)	Mesau_04030 (96)
LpxB	MCHK_2334	Mll0630 (98)	Mesci_3970 (90)	Mesop_4267 (78)	Mesau_04032 (87)
LpxC	MCHK_3169	Mll1545 (98)	Mesci_3157 (93)	Mesop_3548 (96)	Mesau_03428 (97)
LpxD	MCHK_2338	Mll0635 (98)	Mesci_3974 (92)	Mesop_4263 (97)	Mesau_04028 (97)
LpxH	MCHK_2491	Mll0806 (99)	Mesci_4111 (95)	Mesop_4134 (96)	Mesau_03897 (94)
LpxK	MCHK_1590	Mlr8270 (96)	Mesci_4574 (91)	Mesop_5044 (94)	Mesau_04635 (94)
LpxXL	MCHK_2827	Mlr1179 (99)	Mesci_3417 (91)	Mesop_3900 (93)	Mesau_03686 (91)
KdtA	MCHK_1589	Mlr8269 (99)	Mesci_4575 (96)	Mesop_5045 (97)	Mesau_04636 (96)
AcpXL	MCHK_2823	Mlr1174 (99)	Mesci_3421 (99)	Mesop_3904 (99)	Mesau_03690 (99)
KdsA	MCHK_2158	Mlr0374 (99)	Mesci_3689 (96)	Mesop_4551 (97)	Mesau_04186 (96)
KdsB	MCHK_6499	Mlr5497 (98)	Mesci_0064 (94)	Mesop_0064 (96)	Mesau_00066 (93)
HldD	MCHK_4164	Mlr2565 (97)	Mesci_2234 (94)	Mesop_2376 (96)	Mesau_02272 (96)
HldE	MCHK_4162	Mll2562 (99)	Mesci_2238 (94)	Mesop_2378 (95)	Mesau_02276 (94)
GmhA	MCHK_4161	Mll2561 (98)	Mesci_2239 (95)	Mesop_2379 (97)	Mesau_02277 (92)
GmhB	MCHK_4160	Mll2559 (93)	Mesci_2240 (71)	Mesop_2380 (81)	Mesau_02278 (82)
RfaF	MCHK_4165	Mlr2566 (96)	Mesci_2233 (93)	Mesop_2375 (94)	Mesau_02271 (93)
RfaC	MCHK_4166	Mlr2567 (94)	Mesci_2232 (88)	Mesop_2374 (89)	Mesau_02270 (83)
