# Supplementary material for: Whole-genome sequencing of Mesorhizobium huakuii 7653R provides molecular insights into host specificity and symbiosis island dynamics
Source: BMC Genomics. 2014 Jun 6;15(1):440. doi: 10.1186/1471-2164-15-440 (PMC4072884; doi:10.1186/1471-2164-15-440)
Supplement: Supplementary file 4 — Additional file 4: Figures S1 to S5: Figure S1 Nodulation genes participating in NF synthesis. nodE and/or nodF from the 7653R chromosome, pMhu7653Rb, or both participate in synthesis of NFs. Figure S2 Lipopolysaccharide biosynthesis pathway in Mesorhizobium huakuii 7653R. Biosynthesis substrates and products and key enzymes of each step are indicated. Figure S3 ACT visualization of 7653R, R7A, and MAFF303099 chromosomes and plasmids. Genomic alignment of strains 7653R, R7A, and MAFF303099 was performed using ACT [38]. Red connections represent syntenic regions; blue ones represent inversions. The R7A genome with contigs in the order of contigs 1, 2, and 3 is at the top of the figure. The 7653R genome with replicons in the order of Chromosome, pMhu7653Ra and pMhu7653Rb is in the middle and the MAFF303099 genome is at the bottom in the order of Chromosome, pMLa, and pMLb. Figure S4 Genomic islands (GEIs) predicted for the four Mesorhizobium strains by IslandViewer. GEIs are shown for MAFF303099 (A); WSM1271 (B); WSM2073 (C); and WSM2075 (D). Genomes in EMBL or GENBANK format are used. The green ellipse indicates the position of the GEI, which is the same as the symbiosis island on each chromosome. Figure S5 Comparison of codon usage among genomic islands (GEIs) in Mesorhizobium. Codon usage patterns were compared between GEIs and the remaining chromosomes. Lysine codon usages are not included because of the huge variability. (PDF 1 MB) [file 12864_2013_6156_MOESM4_ESM.pdf]

Additional file 4

This file contains 5 supplemental figures, Figures S1-S5.

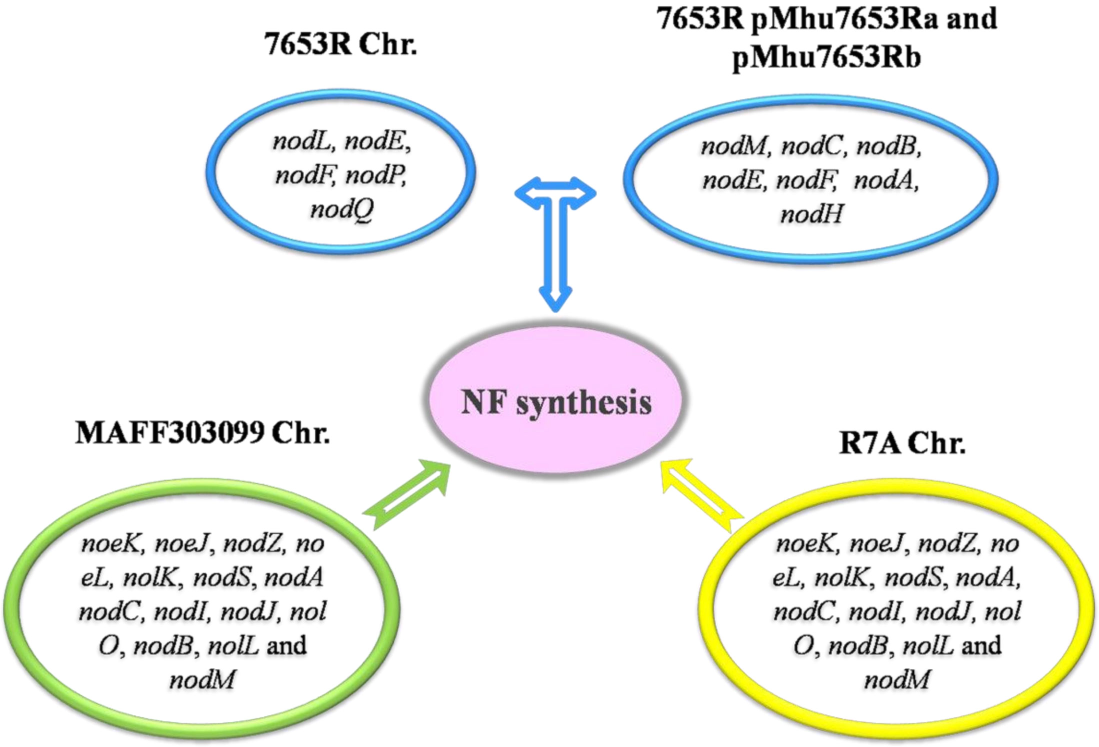

Figure S1 Nodulation genes participating in NF synthesis.

nodE and/or *nodF* from the 7653R chromosome, pMhu7653Rb, or both participate in NF synthesis.

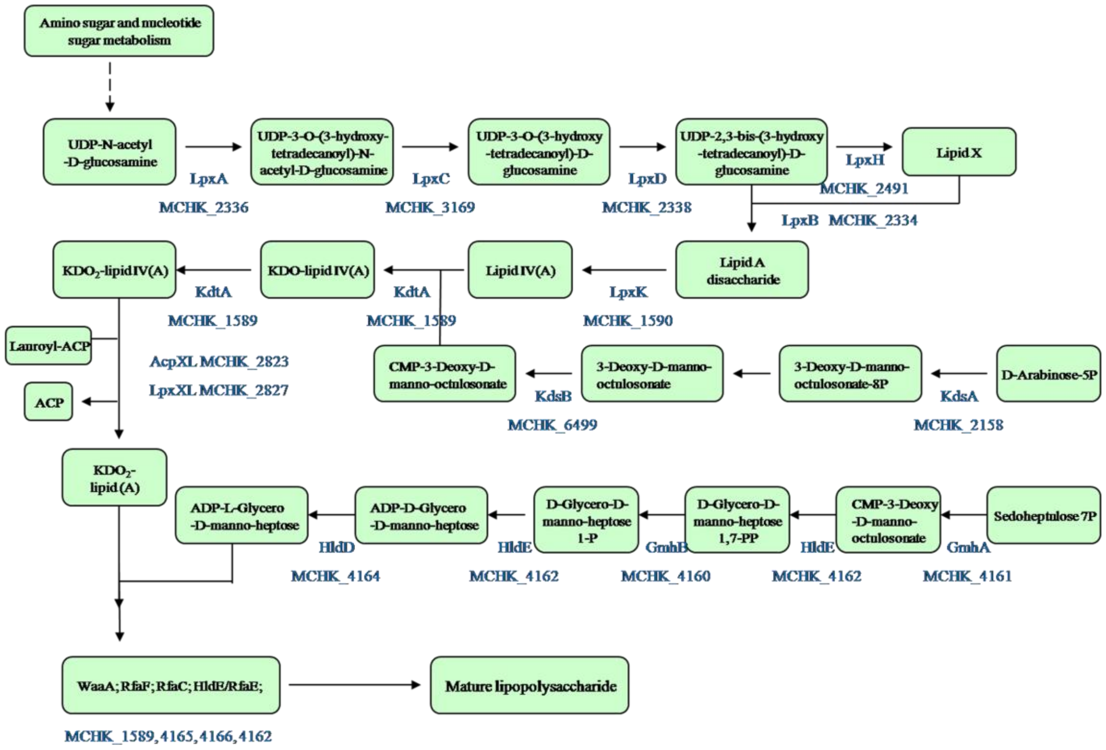

Figure S2 Lipopolysaccharide biosynthesis pathway in *Mesorhizobium huakuii* 7653R.

Biosynthesis substrates and products and key enzymes of each step are indicated.

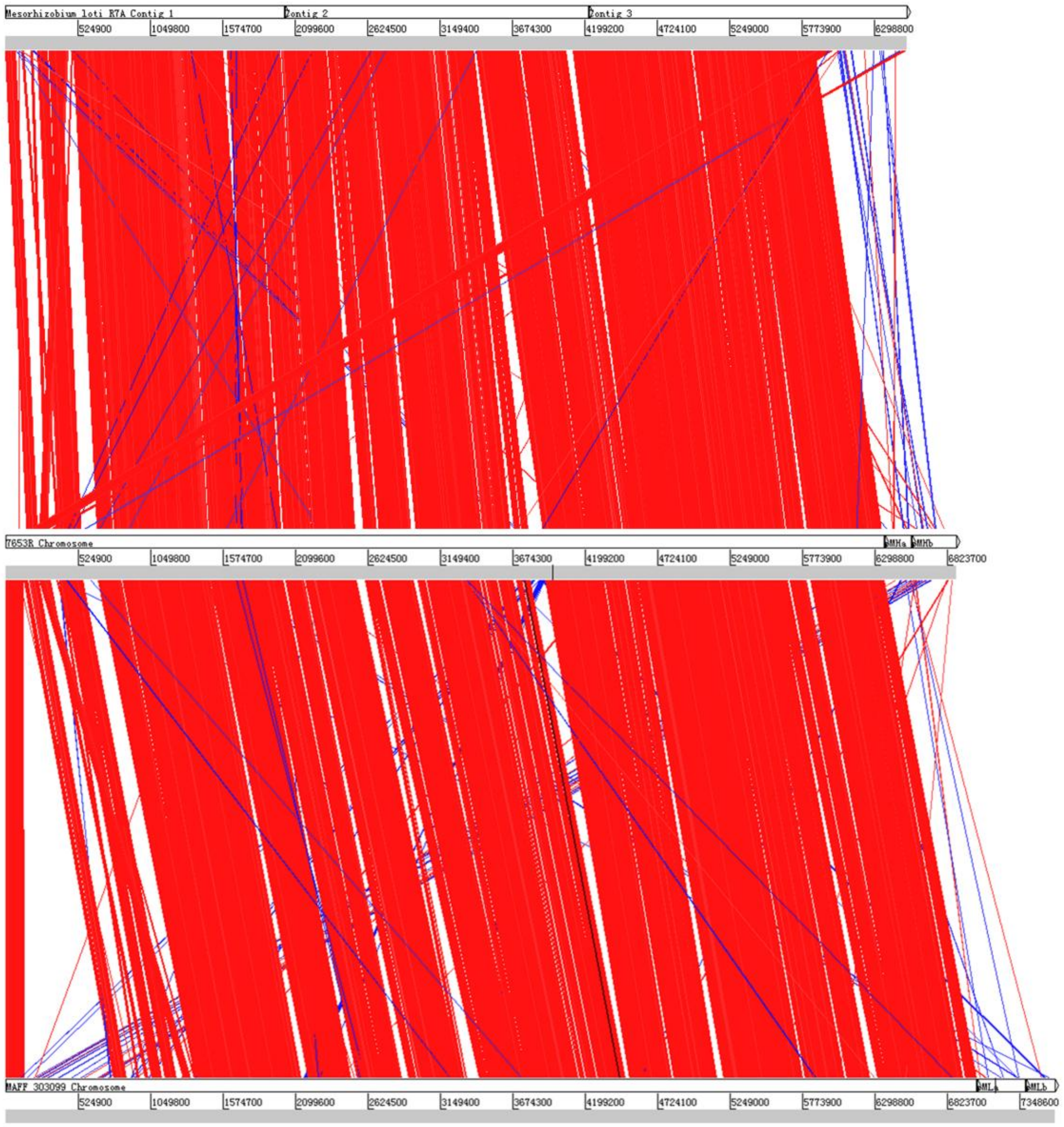

Figure S3 ACT visualization of 7653R, R7A, and MAFF303099 chromosomes and plasmids.

Genomic alignment of strains 7653R, R7A, and MAFF303099 was performed using ACT [37]. Red connections represent syntenic regions; blue ones represent inversions. The R7A genome with contigs in the order of contigs 1, 2, and 3 is at the top of the figure. The 7653R genome with replicons in the order of Chromosome, pMhu7653Ra, and pMhu7653Rb is in the middle, and the MAFF303099 genome is at the bottom in the order of Chromosome, pMLa, and pMLb.

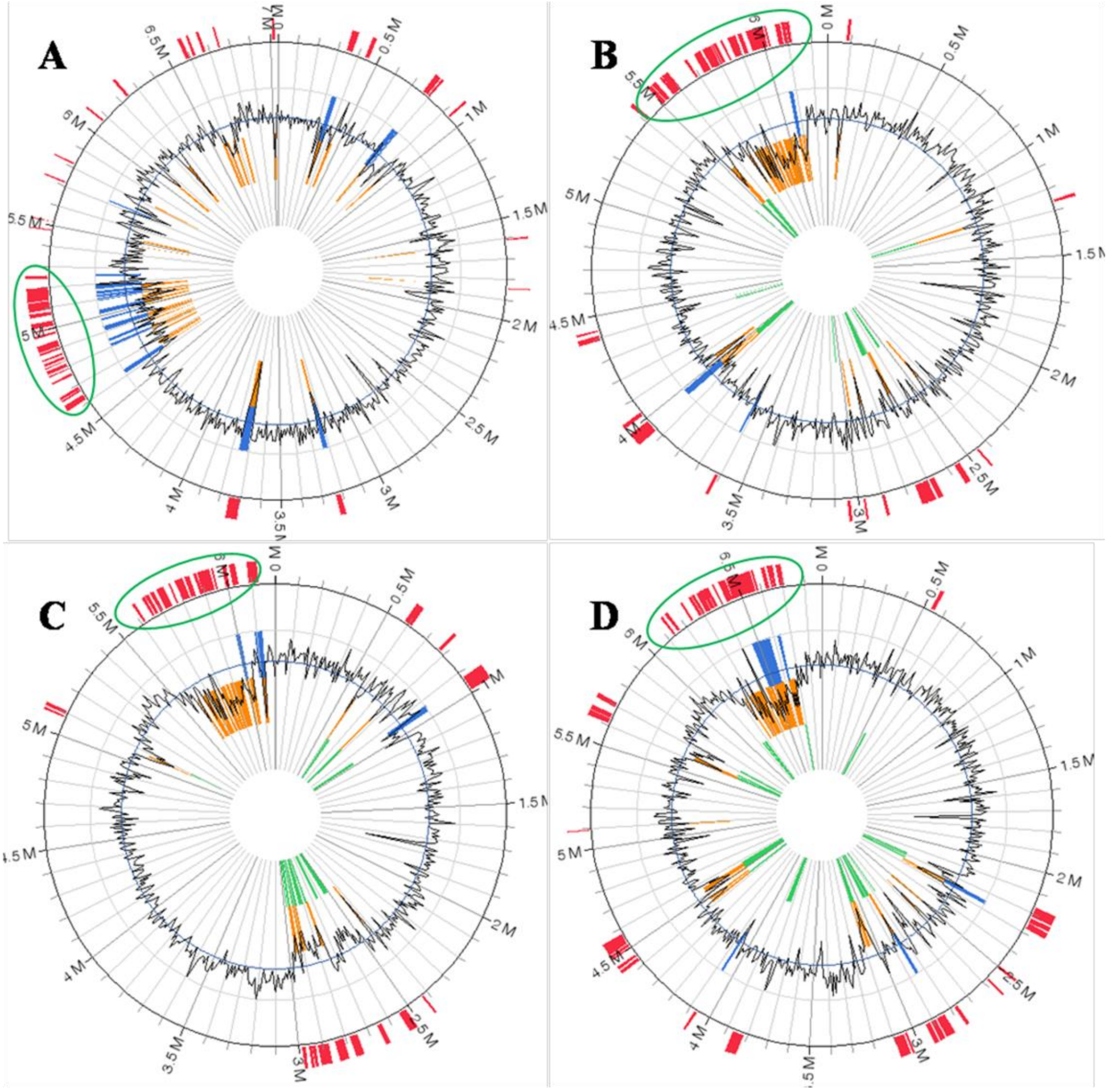

Figure S4 Genomic islands (GEIs) predicted for the four *Mesorhizobium* strains by IslandViewer.

GEIs are shown for MAFF303099 (A), WSM1271 (B), WSM2073 (C), and WSM2075 (D). Genomes in EMBL or GENBANK format are used. The green ellipse indicates the position of the GEI, which is the same as the symbiosis island on each chromosome.

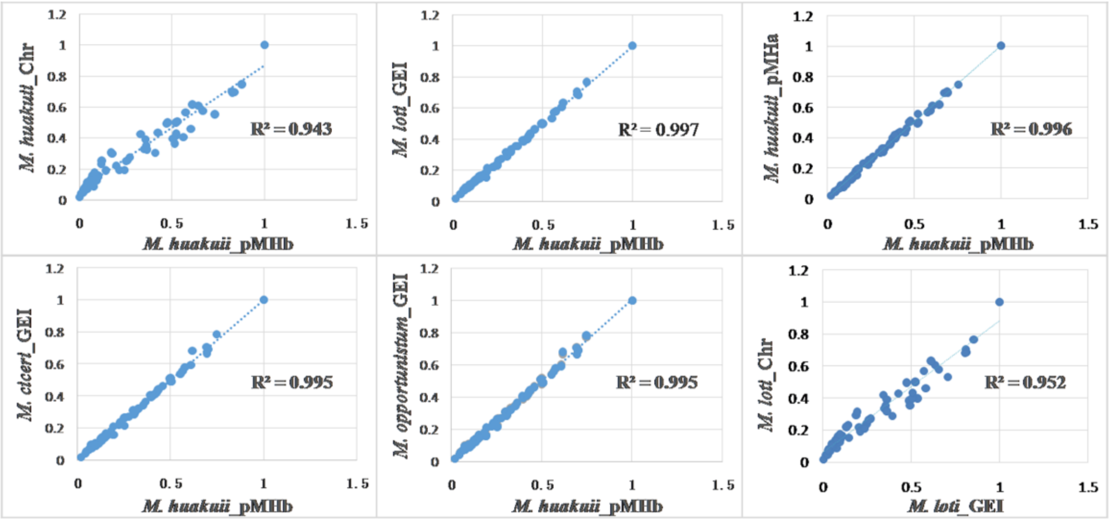

Figure S5 Comparison of codon usage among genomic islands (GEIs) in *Mesorhizobium*.

Codon usage patterns were compared between GEIs and the remaining chromosomes. Lysine codon usage is not included because of the huge variability.
